# Increased TSPO PET signal after radiochemotherapy in IDH-wildtype glioma—indicator for treatment-induced immune activation?

**DOI:** 10.1007/s00259-022-05844-3

**Published:** 2022-05-25

**Authors:** Stefanie Quach, Adrien Holzgreve, Louisa von Baumgarten, Maximilian Niyazi, Marcus Unterrainer, Niklas Thon, Sophia Stöcklein, Peter Bartenstein, Jörg-Christian Tonn, Nathalie L. Albert

**Affiliations:** 1 Department of Neurosurgery, University Hospital, LMU Munich, Marchioninistr. 15, 81377 Munich, Germany; 2 Department of Nuclear Medicine, University Hospital, LMU Munich, Munich, Germany; 3 German Cancer Consortium (DKTK) partner site Munich, Munich, Germany; 4 German Cancer Research Center (DKFZ), Heidelberg, Germany; 5 Department of Radiation Oncology, University Hospital, LMU Munich, Munich, Germany; 6 Department of Radiology, University Hospital, LMU Munich, Munich, Germany


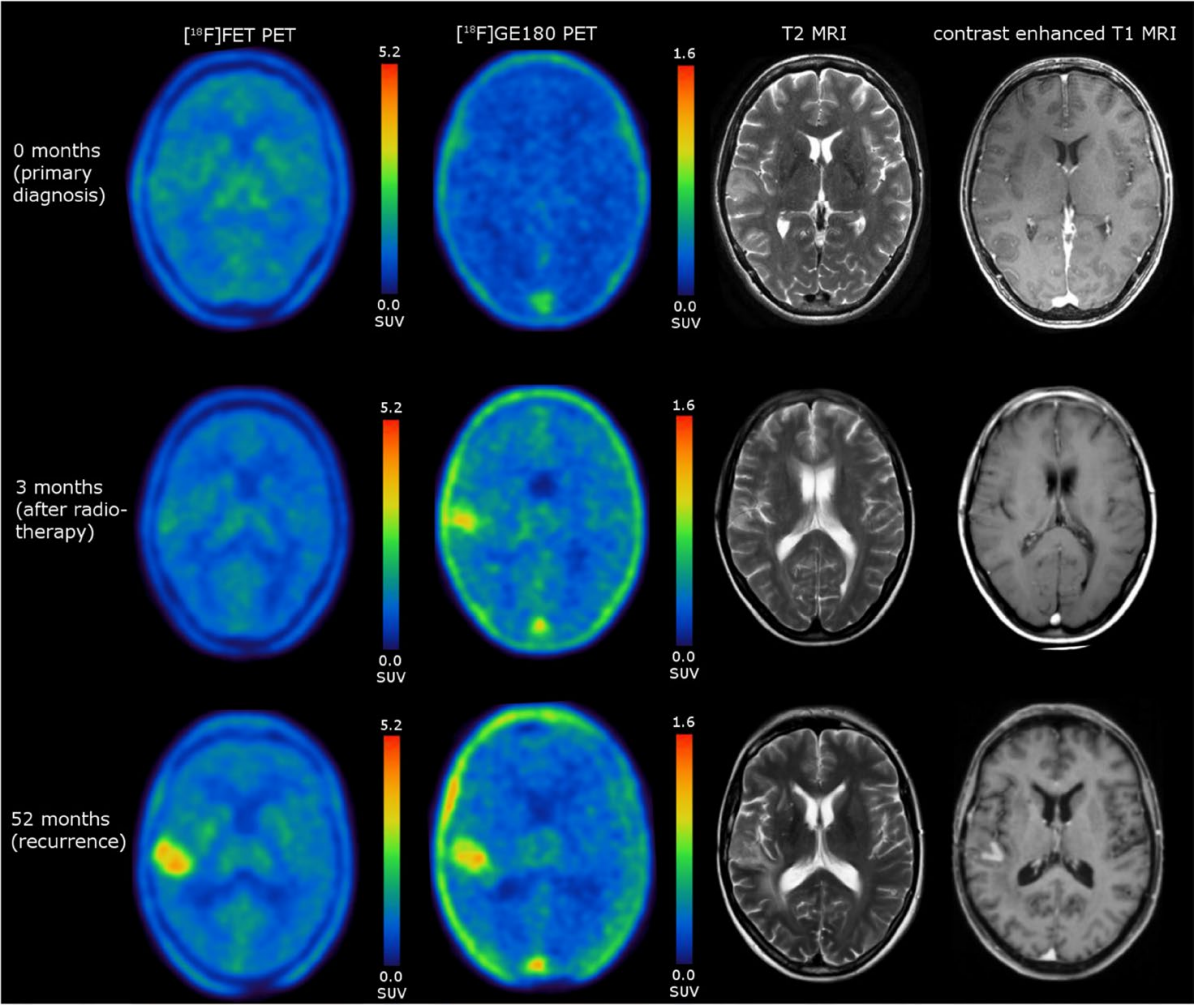


A 51-year-old female patient presented with newly onset seizure. MR imaging revealed a T2-hyperintense lesion without contrast enhancement in the right temporoinsular region showing low uptake on both amino acid PET with [^18^F]FET and TSPO PET with [^18^F]GE180. Stereotactic biopsy revealed an IDH-wildtype glioma, *MGMT*-methylated, with a TERT promoter mutation and a proliferation index of 10%, consistent with a WHO grade 4 glioblastoma according to the current classification [1]. After radiochemotherapy according to EORTC/NCIC protocol [2], [^18^F]FET uptake remained continually low, while [^18^F]GE180 PET showed a focally increased uptake in the treated area. The patient remained stable for a remarkably long period, until tumor recurred locally after 4.3 years. At recurrence, both [^18^F]FET PET and [^18^F]GE180 PET showed an equally strong uptake.

TSPO expression has not only been linked to malignant tumor cells [3–6], but is particularly known as an inflammation marker [7–9]. As such, increased [^18^F]GE180 uptake immediately after therapy in the absence of [^18^F]FET uptake might reflect treatment-related inflammation as has been described after radio- [10, 11] or chemotherapy [12]. This is the first human case demonstrating diverging amino acid and TSPO PET findings after radiochemotherapy in a glioblastoma patient with favorable treatment response. Although low initial FET uptake also indicates favorable outcome and no tissue samples are available for histological correlation immediately after therapy, it is intriguing to speculate that the distinct uptake patterns in dual tracer PET imaging might capture therapy-induced immune response, which may serve as interesting biomarker and should be evaluated in future studies.

## References

[1] Louis DN (2021). The 2021 WHO classification of tumors of the central nervous system: a summary. Neuro Oncol.

[2] Stupp R (2005). Radiotherapy plus concomitant and adjuvant temozolomide for glioblastoma. N Engl J Med.

[3] Cai L, Kirchleitner SV, Zhao D, Li M, Tonn JC, Glass R, Kälin RE. Glioblastoma exhibits inter-individual heterogeneity of TSPO and LAT1 expression in neoplastic and parenchymal cells. Int J Mol Sci. 2020;21(2):612. 10.3390/ijms21020612 PMC7013601 31963507

[4] Unterrainer M (2020). TSPO PET, tumour grading and molecular genetics in histologically verified glioma: a correlative (18)F-GE-180 PET study. Eur J Nucl Med Mol Imaging.

[5] Unterrainer M (2019). Comparison of (18)F-GE-180 and dynamic (18)F-FET PET in high grade glioma: a double-tracer pilot study. Eur J Nucl Med Mol Imaging.

[6] Albert NL (2017). TSPO PET for glioma imaging using the novel ligand (18)F-GE-180: first results in patients with glioblastoma. Eur J Nucl Med Mol Imaging.

[7] Unterrainer M (2018). TSPO PET with [(18)F]GE-180 sensitively detects focal neuroinflammation in patients with relapsing-remitting multiple sclerosis. Eur J Nucl Med Mol Imaging.

[8] Zinnhardt B, Roncaroli F, Foray C, Agushi E, Osrah B, Hugon G, Jacobs AH, Winkeler A. Imaging of the glioma microenvironment by TSPO PET. Eur J Nucl Med Mol Imaging. 2021;49(1):174–85. 10.1007/s00259-021-05276-5 33721063

[9] Zinnhardt B (2020). TSPO imaging-guided characterization of the immunosuppressive myeloid tumor microenvironment in patients with malignant glioma. Neuro Oncol.

[10] Deloch L (2016). Modern radiotherapy concepts and the impact of radiation on immune activation. Front Oncol.

[11] McLaughlin M (2020). Inflammatory microenvironment remodelling by tumour cells after radiotherapy. Nat Rev Cancer.

[12] Foray C (2021). Imaging temozolomide-induced changes in the myeloid glioma microenvironment. Theranostics.

